# Automatic Assistance to Cognitive Disabled Web Users via Reinforcement Learning on the Browser

**DOI:** 10.1007/978-3-030-58805-2_8

**Published:** 2020-08-12

**Authors:** Tomas Murillo-Morales, Peter Heumader, Klaus Miesenberger

**Affiliations:** 8 Institute Integriert Studieren, JKU Linz, Linz, Austria; 9 Jack Baskin School of Engineering, UC Santa Cruz, Santa Cruz, CA USA; 10 Dipartimento di Meccanica, Politecnico di Milano, Milan, Italy; 11 Support Centre for Students with Special Needs, Masaryk University Brno, Brno, Czech Republic; Institute Integriert Studieren, Johannes Kepler University, Altenbergerstraße 69, 4040 Linz, Austria

**Keywords:** Cognitive accessibility, Affect detection, Assistive technology

## Abstract

This paper introduces a proof of concept software reasoner that aims to detect whether an individual user is in need of cognitive assistance during a typical Web browsing session. The implemented reasoner is part of the Easy Reading browser extension for Firefox. It aims to infer the user’s current cognitive state by collecting and analyzing user’s physiological data in real time, such as eye tracking, heart beat rate and variability, and blink rate. In addition, when the reasoner determines that the user is in need of help it automatically triggers a support tool appropriate for the individual user and Web content being consumed. By framing the problem as a Markov Decision Process, typical policy control methods found in the Reinforcement Learning literature, such as Q-learning, can be employed to tackle the learning problem.

## Introduction

### Cognitive Accessibility on the Web

Accessibility to the digital world, including the Web, is increasingly important to enable people with disabilities to carry out normal lives in the information society, something that has been acknowledged by the United Nations and many individual governments to be a right for people with disabilities. This is as true for people with cognitive, language, and learning differences and limitations as it is for anyone else [6]. Nowadays, many Web users suffering from a cognitive or learning disability struggle to understand and navigate Web content in its original form because of the design choices of content providers [6]. Therefore, Web content often ought to be adapted to the individual needs of the reader.

Currently available software tools for cognitive accessibility of Web content include Immersive Reader [4], the Read&Write browser extension [13], and Easy Reading [3]. These tools embed alternative easy-to-read or clarified content directly into the original Web document being visited when the user requests it, thereby enabling persons with a cognitive disability to independently browse the Web. Access methods may be tailored to the specific users based on personal data, generally created by supporting staff or educators [8]. Besides these semi-automatic tools, current approaches to making websites accessible to people with cognitive and learning impairments still mostly rely on manual adaptations performed by human experts [8].

### The “Easy Reading” Framework

The Easy Reading framework^1^ improves cognitive accessibility of original websites by providing real time personalization through annotation (using e.g. symbol, pictures, videos), adaptation (e.g. by altering the layout or structure of a website) and translation (using e.g. Easy-to-Read, plain language, or symbol writing systems) [3].

The main advantage of the Easy Reading framework over existing cognitive support methods is that the personalized support tools are provided at the original websites in an automatic fashion instead of depending on separate user experiences which are commonly provided to users in a static, content-dependent manner and that must be manually authored by experts.

**Fig. 1.**
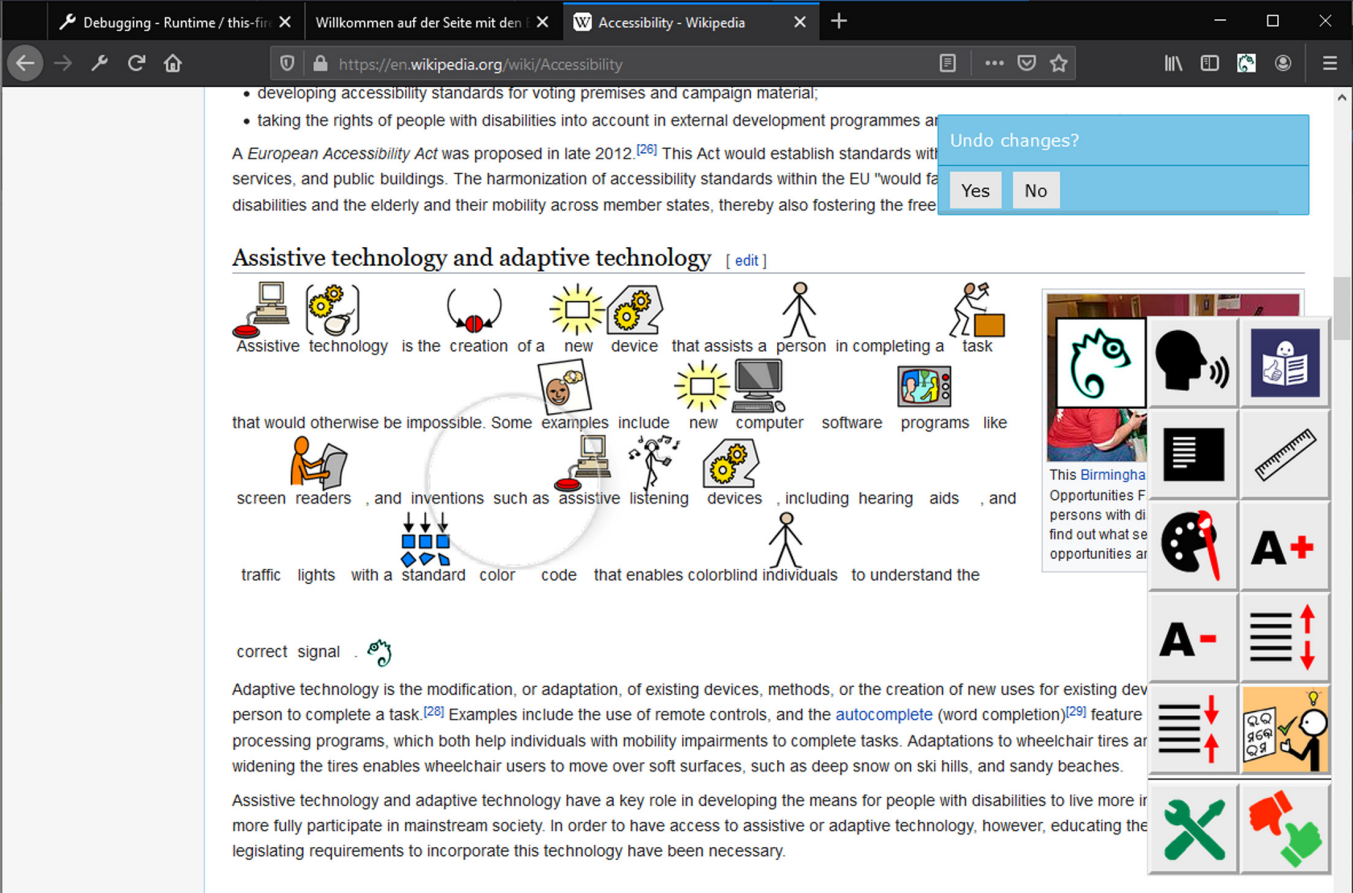
The Easy Reading graphical user interface (GUI) overlaid on a website. The symbol support tool has been automatically triggered on a text paragraph by the Easy Reading reasoner, adapting its content automatically with symbol annotations over the original text. The user may reject automatically given help by means of an onscreen dialogue (top right). Any of the available tools on the Easy Reading GUI may be also manually triggered by the user at any given time by clicking on its corresponding button.

Easy Reading software clients have been designed as Web browser extensions^2^ (for Mozilla Firefox and Google Chrome) and mobile OS apps (Android and iOS). The main interaction mechanism between the user and the client consist on a graphical user interface (GUI) that the user may choose to overlay on top of any Website being currently visited. A number of tools, personalized to the specific user, are available to the user in Easy Reading’s GUI (see Fig. 1). The user may choose at any time to use some of the available framework functions by triggering their corresponding tool by clicking on the available buttons of the GUI.

### Problem Statement

Given the special needs of Easy Reading’s user base, having a traditional GUI as the only interaction mechanism between the user and the browser extension may not suit the specific needs of all users. Some users, especially those suffering from a profound cognitive disability, may not possess the necessary expertise and/or understanding to interact with Easy Reading’s GUI. This is particularly the case if there are many tools being overlaid on the GUI, as this may overwhelm the user given the considerable amount of personalization mechanisms to choose from. The use of Easy Reading is also restricted for those suffering from additional physical disabilities making interaction slow or impossible when no easy to use AT solutions are at hand.

**Fig. 2.**
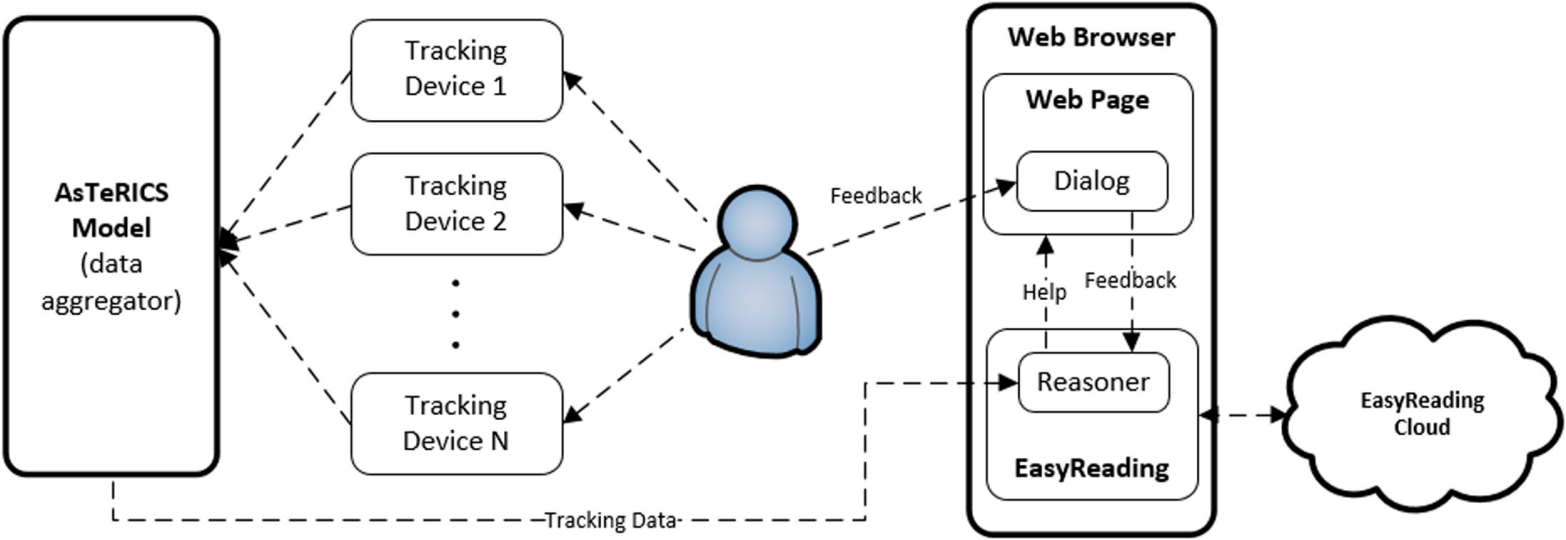
Main components of the Easy Reading User Tracking and Reasoning Framework

We therefore aim to assist the user in choosing and using the right cognitive support tool when he or she is in need of help while navigating Web content which appears to be confusing or unclear. We have expanded the Easy Reading framework so that it supports the automatic triggering of any support tool with the addition of two components; namely, (1) a user data collection module and (2) a client-based reasoner that learns about the mental state of the user based on the gathered data and previous experiences, and reacts accordingly by triggering support tools when necessary. Figure 2 displays the interaction between these two components within the Easy Reading framework.

The next section gives a short overview on current methods for automatically detecting the cognitive load/affect of a person from collected user data. Based on some of these results, the design of the Easy Reading User Tracking and Reasoning framework is outlined in the remaining of this document.

## Affect Detection: State of the Art

Affect recognition is the signal and pattern recognition problem that aims to detect the affective state of a person based on observables, with the goal of, for example, providing reasoning for decision making or supporting mental well-being [14]. Terms such as affect and mood elude a precise definition in the literature, but some working definitions may be characterized. Namely, **affect** is a neurophysiological state that is consciously accessible as the simplest raw, nonreflective, primitive feeling evident in mood and emotions e.g. the feeling of being scared while watching a scary movie [7]. On the other hand, **emotions** are intense and directed indicators of affect e.g. shock and scream are emotions that indicate the affect of being scared [7]. As opposed to emotions, **moods** are less intense, more diffuse, and last for a longer period of time than emotions [14]. For example, the emotion of anger, which does not last long by itself, can lead to an irritable mood [14]. On this paper we focus on the binary classification problem of the user’s affect, namely, whether the user is in a confused mental state during a Web browsing activity. This problem is closely related to that of stress detection, in which data is analyzed to predict the stress level of a person, generally as a binary variable (stressed/unstressed). Stress detection is a well-researched problem that can be reliably undertaken by analyzing user physiological signals provided by e.g. a wrist-worn device such as a smartwatch [16].

Affect is a subjective experience of a person which is generally detected through self-reporting. Nevertheless, numerous approaches that aim to infer a person’s affective state in an automatic fashion can be found in the literature. These approaches can be divided into four categories depending on the kind of data they process:

- Contextual approaches learn from the interaction between the user and a software system by e.g. analyzing mouse gestures or page visit times.
- Physiological approaches collect and analyze physiological data from the user, such as heart beat rate or skin temperature.
- Text-based approaches process and interpret the textual contents of speech spoken or written by the user using natural language processing (NLP) techniques for sentiment analysis generally based on supervised machine learning methods.
- Audio-visual approaches study recorded audio (generally speech) or video (of e.g. the user’s face or full body) while the user interacts with the system.

Preferably, affect recognition systems should employ multimodal data i.e. fusion analysis of more than one input modality, since multimodal affect recognition system are consistently more accurate than unimodal methods [1]. A collection of state-of-the-art methods for affect recognition can be found in [1, 12, 14]. The vast majority of these methods rely on supervised machine learning models such as Deep Neural Networks (DNN) for image analysis e.g. for analyzing the user’s facial expressions; or Random Forests (RF) and Support Vector Machines (SVM) for analysis of physiological signals e.g. heart rate variability. What these methods have in common is that they require of big amounts of training data that the learning model must be trained on. Available datasets such as the well-known DEAP dataset [5] aim to simplify this process by providing a large amount of pre-labelled training data for affect recognition tasks. For a list of available datasets the reader is directed to [12] and [14]. However, these approaches, especially those relying on physiological signals, suffer from a number of drawbacks that hinder their application in practice:

- Even if supervised models perform well when tested on known users, they exhibit high generalization errors when tested on unknown users, and thus models must be fine-tuned to the specific user [9].
- Available datasets have been collected using a specific combination of devices and sensors e.g. a specific wristband. Therefore, end users are forced to acquire a very similar combination of devices to make use of models trained on such datasets. Preferably, the reasoner model should adapt to the available hardware, not the other way around.
- Many tracking devices employed to collect data for these datasets are too expensive or obtrusive to be used in an informal home/office setting by end users, such as EEG headsets.

## Easy Reading User Tracking and Reasoning

This section introduces our approach to automatic affect detection tailored to the specific user and available devices that aims to overcome some of the issues described in the previous section.

### User Tracking Data Collection

In order to detect whether the user is confused or mentally overwhelmed by the content he or she is visiting during an ordinary Web browsing activity, user data needs to be collected in a transparent, unobtrusive manner to the user. In addition, specific tracking devices whose presence in a common household or office environment would normally be unwarranted (due to e.g. high cost) ought to be avoided. Therefore, after a study of the relevant literature and filtering out those tracking devices which did not satisfy these requirements, the following signals were considered:

- **Eye movement and position**. The current position of the user’s gaze on the screen and the voluntary or involuntary movement of the eyes can be collected with the use of inexpensive eye trackers, commonly used in gaming, that can be mounted near a computer’s screen in close distance to the user. Eye movement data is of great utility to ascertain cognitive load. Some authors even argue that eye movement data suffices to infer the cognitive demand of tasks being carried out by a person [15].
- **Blink rate**. The time period between two or more consecutive eye blinks can be a good indicator of task difficulty as well. For example, a poor understanding of the subject matter in a lecture on mathematics resulted, for some persons, on an increased number of rapid serial blinks [10]. Blink frequency can be easily measured by, for example, analysing a video of the user’s face recorded with a typical laptop webcam.
- **Heart Rate**. The current heart rate (HR) of the user, measured in beats per minute (BPM), and especially heart rate variability (HRV), which describes the variation of the time between heartbeats, is a rather simple but effective measure of the current affective state and stress level of the user [14]. These dimensions can be easily determined with the use of commercial smartwatches and fitness trackers, which are the most popular wearable devices being sold nowadays.
- **Implicit behavioural information**. Several measures of user behaviour on websites that aim to predict disorientation, task difficulty and user preferences can be found in the information retrieval literature. For example, time spent on site and click-through rates have been used to measure the cognitive load of users visiting a web search engine [2]. It is however important to note that other studies have concluded that user disorientation on websites is only weakly related to user behaviour [2]. Therefore, physiological signals are employed as the main data source employed by Easy Reading’s reasoner module.

User data are processed and gathered by the Easy Reading framework as follows. Eye fixation duration (in milliseconds) and current x and y coordinates of the user gaze on the screen is measured by a Tobii 4C Eye Tracker^3^. To measure HR and HRV, the Vivoactive 3 smartwatch^4^ by Garmin was selected as a good compromise between accuracy and affordability. Blink rate can be measured from video data recorded from any standard webcam, whether integrated in a laptop or an external one. Input signals are gathered and processed in an easy, flexible, and tailorable manner by means of an AsTeRICS model.

AsTeRICS [11] is an accessible technology (AT) construction set that provides plug-ins for many common input devices and signal processing operations. By combining already existing and newly developed plug-ins into an AsTeRICS model, raw input physiological signals are pre-processed before being sent to the reasoning module. Pre-processing includes methods for synchronization of data streams, handling of missing values (e.g. if the user does not possess some of the input devices), noise removal, and segmentation of the collected data into batches. Several pre-processing parameters can be adjusted directly in the AsTeRICS model by end-users or carers without the need of possessing technical skills. For example, batch (temporal window) size is by default set to 120 s (e.g. 12 samples aggregated after 10 s each) following state-of-the-art recommendations [16], but can be easily adjusted by modifying the relevant parameters of the Easy Reading AsTeRICS Data Collector plug-in. Collected batches are next converted to JSON objects and sent to the Easy Reading browser extension via a secure WebSocket connection maintained by the AsTeRICS Runtime Environment (ARE) Web server.

### Easy Reading Reasoner

The Easy Reading Reasoner is the client-based module in charge of solving the problem of inferring the affective state of the user from the current readings of physiological signals collected by a running AsTeRICS model. The reasoner is hosted on the client in order to minimize the amount of messaging needed between the distributed components of the user tracking and reasoning framework, which in turn results in more responsive reasoner actions. This however comes at the cost of a more limited computational capacity, as the whole learning model has to run on the user’s browser.

We have adopted a Markov decision process (MDP) as the framework for the problem, which allows it to be theoretically solved using a number of well-established control learning methods in the reinforcement learning (RL) literature. As previously stated, research shows that affection/stress recognition methods must be tailored to the individual differences of each person. Given that RL is specially well suited to problems in which the only way to learn about an environment is to interact with it, we model the environment shown in Fig. 2 as a MDP to be solved for a specific user. For a detailed characterization of MDPs and RL, the reader is directed to [17], Chapters 1 and 3. Like every MDP, our problem consists of an agent, (intractable) environment, state set (***S***), action set (***A***), and policy (*π*), characterized as follows.

The agent in a MDP is the learner and decision maker. It corresponds to the reasoner module being executed in the background script of the browser extension, as shown in Fig. 2. At any given time step, $t$, the reasoner observes the current state of the user, $s_{t}$, as specified by each sample being delivered by the AsTeRICS model, and decides on an action, $a_{t}$, to be taken with probability $p$ i.e. $\pi \left( {a_{t} |s_{t} } \right) = p$. The current status, $s_{t}$, is the JSON object produced by the data collector model, which consists on a number of features e.g. HRV, and the current value of the user readings for that feature. Note that $t$ does not correspond to an exact moment in time, but rather to the latest time window that has been aggregated by the data collector. Depending on the feature, the data collector sets its value to the latest received input or an aggregation thereof, such as the average or most common value (mode) during the time window.

The reasoner may next take one of three actions ($a_{t}$), namely:

1. No action (**NOP**). The reasoner has inferred that the user is not in need of help at the moment, and thus no further action is necessary.
2. Help user (**Help**). The reasoner has inferred that the user is in need of help with some content of the website being currently visited, and a suitable framework tool needs to be triggered. Figure 1 displays an example of this action being triggered on a website.
3. Ask user explicitly for the next action to take (**Ask**). The reasoner is unsure about the next action to take, as it expects both NOP and help actions to yield a low reward. In this case, it asks the user, via an onscreen dialogue, about which of these two actions to take next.

The user gives feedback, which may be implicit or explicit, on the action just taken by the reasoner and a numeric reward, $r_{t + 1}$, is computed as a function of $a_{t}$ and the user feedback, as shown in Table 1. This reward function heavily penalizes the case in which the agents fails to help a user in need. However, to prevent the agent from persistently asking the user for explicit feedback on the best action to take, asking the user is always given a (low) negative reward as well. Moreover, since the correct $\left( {s_{t} , a_{t} } \right)$ pair is known after the user has answered a feedback dialogue, this combination is accordingly rewarded in order to speed learning up. The agent is only positively rewarded in the case that it manages to predict that the user is currently in need of help and automatically triggers the correct tool for his or her needs.

**Table 1.** Reward function of the Easy Reading reasoner

$a_{t}$	Implicit user feedback		Explicit user feedback			
	User does not react/browses as usual for a given time	User manually triggers a tool	User accepts automatic help	User rejects automatic help	User requests help in dialog	User rejects help in dialog
No action	0	−200	*N/A*	*N/A*	*N/A*	*N/A*
Help user	+10	*N/A*	+10	−20	*N/A*	*N/A*
Ask user	−10	−10 (+10 to $a_{t}$ = help user)	*N/A*	*N/A*	−10 (+10 to $a_{t}$ = help user)	−10

Currently, the support tool triggered by the reasoner is the most frequently used tool by the user for the content type (text or image) that has been stared at the longest during the last time window $t$. Use frequency for each tool is computed and stored in each user’s profile on Easy Reading’s cloud backend.

**Fig. 3.**
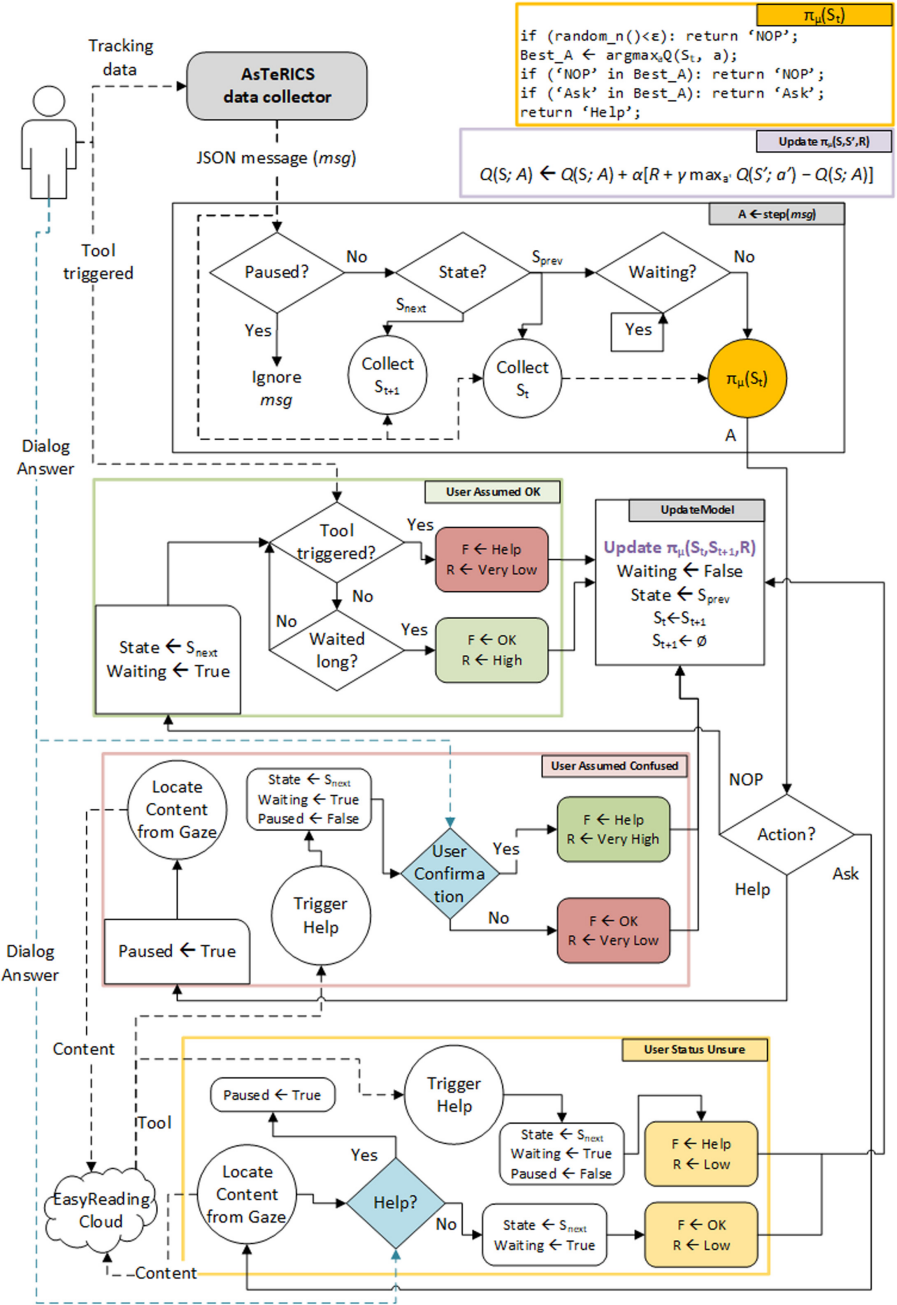
Simplified Easy Reading Reasoner Workflow

In the subsequent time step, $t + 1$, the agent receives, along with the reward signal $r_{t + 1}$, a new user state, $s_{t + 1}$. This state is an aggregation of user data representing the user’s reaction to $a_{t}$. Therefore, the Easy Reading extension does not start collecting a new state until the user’s feedback has been gathered. Consequently, any user data incoming during the processing of $s_{t}$, and after $a_{t}$ has been yielded but before the user feedback has been inferred, is discarded. This whole process is summarized in the workflow diagram shown in Fig. 3.

The goal of the agent (reasoner) is to learn which sequence of actions leads to a maximum reward in the long run. This behavior is encoded in a so-called policy, which the reasoner has to learn. The policy, $\pi \left( {a|s} \right)$, specifies which action to take on a given state, or, in the nondeterministic case, the probability of each action of the action space for a given state.

The Easy Reading reasoner keeps an estimation of the value, in terms of future expected returns, of each action $a$ performed on each state $s$ it has seen so far, $q_{\pi } \left( {s,a} \right)$, known as the action-value function of $\pi$. This function can be stored as a table in memory if the set state is small. In this case, input observables are further pre-processed in the browser extension via data binning to obtain a manageable state set. Otherwise, the state-value function can be approximated via function approximation methods e.g. by modelling it with a neural network (NN). The latter approach is possible by defining actor and/or critic NNs with Tensorflow.js directly on the user’s browser^5^. Note however that due to the strict security policy of the Firefox add-on store this possibility cannot be included in officially listed extensions, and therefore our extension currently only implements a tabular q-function.

The policy that maximizes $q_{{\pi^{*} }} \left( {s,a} \right)$, for all states and actions is known as the optimal policy, $\pi^{*}$. Some RL control methods, such as Temporal-Difference (TD) learning methods, converge to the optimal policy given enough training time. The Easy Reading reasoner implements a number of value-based RL methods that aim to find $\pi^{*}$; namely, Q-learning and double-Q-learning. Describing these methods is out of scope of this paper, for further information the reader is directed to [17]. The basic q-learning update rule is shown in context in Fig. 3.

When interacting with a new user, the agent does not know anything about the environment (the user), and therefore it has to be explored. Once enough knowledge is acquired, this knowledge can be exploited to maximize the rewards obtained from this point onwards. The agent follows a ε-greedy behavioral policy with respect to $q_{\pi } \left( {s,a} \right)$, whose values are initialized to zero. However, instead of choosing a random action with ε probability, it chooses the “no action” action both at exploration time and when state-action values are tied at exploitation time. This way, since most of the time the user will not be in a confused state, help tools and dialogues are less likely to come up at unsought times, which aims to reduce user frustration overall. The system can then slowly learn to identify the states in which the user needs help as tools are manually triggered during training sessions with a caregiver where negative feedback is acquired. The implemented policy’s pseudocode is shown in context in Fig. 3.

## Conclusions and Future Work

This article has introduced an innovative approach to automatically detecting the affective state of a web user in real time based on the analysis of physiological signals. The main goal of the Easy Reading reasoner is to infer the current cognitive load of the user in order to automatically trigger the corresponding assistance mechanism of the Easy Reading framework that would help the user in getting a better understanding of a difficult piece of Web content (text or image). It is included in the latest version of the Easy Reading extension as a proof of concept and is ready to be tested with voluntary participants.

Training sessions with synthetic data have been carried out, yielding a very good accuracy of around 90% after 150 time steps i.e. around 5 h of real training time. However, detecting user confusion on actual users may prove much more challenging, since changes in physiological signals may be too subtle or complex to be properly modelled by the Easy Reading reasoner and the inexpensive consumer devices employed. It must be noted that initial informal evaluation with end users has shown that triggering support tools when the user does not need them should be avoided altogether, since it frustrates and confuses them to the point where they refuse to keep using the Easy Reading extension with reasoner support enabled. After this observation, we modified the agent’s policy from a traditional ε-greedy policy to the modified policy shown in Fig. 3. The next step is to test our approach with end users in a laboratory setting.

## Footnotes

1 https://www.easyreading.eu/.

2 We recommend downloading the Easy Reading extension for Firefox at the following URL: https://addons.mozilla.org/en-US/firefox/addon/easy-reading-for-firefox/.

3 https://gaming.tobii.com/tobii-eye-tracker-4c/.

4 https://buy.garmin.com/en-US/US/p/571520/.

5 https://www.tensorflow.org/js.
